# Hybrid SFNet Model for Bone Fracture Detection and Classification Using ML/DL

**DOI:** 10.3390/s22155823

**Published:** 2022-08-04

**Authors:** Dhirendra Prasad Yadav, Ashish Sharma, Senthil Athithan, Abhishek Bhola, Bhisham Sharma, Imed Ben Dhaou

**Affiliations:** 1 Department of Computer Engineering and Applications, GLA University, Mathura 281406, Uttar Pradesh, India; 2 Department of Computer Science and Engineering, Koneru Lakshmaiah Education Foundation, Vaddeswaram 522502, Andhra Pradesh, India; 3 Chitkara University School of Engineering and Technology, Chitkara University, Himachal Pradesh 174103, India; 4 Department of Computer Science, Hekma School of Engineering, Computing and Informatics, Dar Al-Hekma University, Jeddah 22246-4872, Saudi Arabia; 5 Department of Computing, University of Turku, 20500 Turku, Finland; 6 Higher Institute of Computer Sciences and Mathematics, Department of Technology, University of Monastir, Monastir 5000, Tunisia

**Keywords:** bone fracture, hybrid, CNN, canny, fusion, classification, X-ray

## Abstract

An expert performs bone fracture diagnosis using an X-ray image manually, which is a time-consuming process. The development of machine learning (ML), as well as deep learning (DL), has set a new path in medical image diagnosis. In this study, we proposed a novel multi-scale feature fusion of a convolution neural network (CNN) and an improved canny edge algorithm that segregate fracture and healthy bone image. The hybrid scale fracture network (SFNet) is a novel two-scale sequential DL model. This model is highly efficient for bone fracture diagnosis and takes less computation time compared to other state-of-the-art deep CNN models. The innovation behind this research is that it works with an improved canny edge algorithm to obtain edges in the images that localize the fracture region. After that, grey images and their corresponding canny edge images are fed to the proposed hybrid SFNet for training and evaluation. Furthermore, the performance is also compared with the state-of-the-art deep CNN models on a bone image dataset. Our results showed that SFNet with canny (SFNet + canny) achieved the highest accuracy, F1-score and recall of 99.12%, 99% and 100%, respectively, for bone fracture diagnosis. It showed that using a canny edge algorithm improves the performance of CNN.

## 1. Introduction

Bone is an essential component of the human body. It helps people to move from one place to another place. A bone fracture may happen due to an accident or other reason. After a fracture, treatment is necessary as early as possible. An orthopedic surgeon examines an X-ray or CT-scan image to detect fractured bone. After that, treatment is given to the patient. The treatment success depends on an orthopedic surgeon’s experience and expertise. This manual process is time consuming and the availability of experts in a remote area is less. In recent days, diagnosis of real-time medical problems is done by machine learning and deep learning technique [1,2]. In addition, several deep CNN models were reported in the past to demonstrate its effectiveness for various applications [3,4].

In the current research work, we aim to detect the bone fracture with a high level of accuracy so that the proper identification of the fracture can be performed and the severity of the bone fracture can be judged. The X-ray images are more sensitive to noise that can be removed using the image processing technique. Several pieces of the literature are available for the analysis of different types of human bones. In this regard, Lu et al. [5] designed a universal fracture detection system using the deep CNN techniques. First, the quality of an image is improved using preprocessing techniques. After that, data augmentation is used to enlarge the size of the dataset. Finally, Ada-ResNeSt is used for the classification of fractured and healthy bone with a mean precision of 68.4%. In other research, Guan et al. [6] enhanced their model performance on the arm bone X-ray images. They designed a model for fracture detection in arm bones. Three factors are included in the core changes. First, to gain more fractural details, a new back-bone network is developed based on the function pyramid architecture. Second, to improve the contrast of original images, an image preprocessing method involves an opening procedure and pixel value transformation.

All the methods discussed above and in Section 2 have done remarkable work in classifying the fractured bone. However, most research is based on machine learning techniques requiring handcrafted training features. This manual feature extraction method requires expertise and classification performance is not optimal. Several pieces of research have been reported based on the transfer learning approach, but their performance is not optimal. In this study, we have trained and analyzed the performance of the AlexNet, VGG16, ResNeXt and MobileNetV2 on the X-ray image of the bone image dataset. The performance of these models is remarkable for the healthy bone, but lacks in the fracture bone diagnosis. The major contribution of this research is we have designed a new multimodal feature fusion-based hybrid SFNet model that takes less computation time, and efficiency is high compared to other state-of-the-art CNN models. The grey images and their corresponding canny image are fed to the model for training and validation. Furthermore, hybrid SFNet + canny achieved the highest classification accuracy. It showed that using a canny edge algorithm improves the performance of CNN. 

### 1.1. Problem Statement

Bone is an essential component of the human body. It helps people to move from one place to another place. A bone fracture may happen due to an accident or other reason. After a fracture, treatment is necessary as early as possible. An orthopedic surgeon examines an X-ray or CT-scan image to detect fractured bone. The diagnosis process for fractured bone requires expertise. Hence, a robust and efficient automated system needs to be designed that can diagnose bone fractures in real-time to assist an orthopedic surgeon. In the past, several research studies were conducted to design an automated system using ML and DL. The ML technique required hand-crafted features due to this optimal solution not being available. In contrast, the DL method extracts features automatically and provides an enhanced diagnosis, but its computation cost is high and requires a large dataset to train the model. 

### 1.2. Motivation

In recent days, the biomedical domain has been tuned with the machine learning and deep learning models. Bone diagnosis using artificial intelligence techniques has been used in past research. These methods have done a tremendous job, but lack in efficiency, and computation cost is high. In the current work, a novel hybrid SFNet has been developed which is highly efficient and the computation cost is less. The hybrid SFNet is a two-scale DL model, which has less neurons, due to this computation cost being less and the high efficiency that is achieved by the training of the model at two scales. First, localization of fractured bone is performed using an improved canny edge detection technique. After that, grey image and their corresponding canny image are fed into hybrid SFNet for deep feature extraction. This process significantly improves the bone fracture diagnosis. 

The major contribution of the proposed study is as follows.

(1) Designed a novel hybrid feature fusion-based SFNet for bone diagnosis.
(2) The canny edge image improves the performance.
(3) The experiment is conducted on a publicly available dataset.
(4) The proposed model classification performance for fractured bone is highest.

### 1.3. Organization of the Research

Section 2 describes the overview of the past research on bone diagnosis. In Section 3, the architecture of different deep CNN models has been discussed, and in Section 4, the proposed model architecture is explained. The comparisons of results are described in Section 5. In Section 6, a summary of the current research work is discussed. Finally, the proposed method is concluded in Section 7.

## 2. Related Work

Basic machine learning techniques, such as preprocessing and feature extraction, has been used in this study [7]. The relaxed digital straight line (RDSS) approach for restoring the false contour to overcome the error that occurs at the time of segmentation in the contour is elaborated in [8]. The same author, Basha et al. [9] developed a model based on the machine learning technique for bone fracture diagnosis. Firstly, the Gaussian filter is used to improve the quality of an X-ray image. Then, the edge is detected using the canny edge algorithm. Finally, the Harris corner detection method is used to identify the fractured regions. This method identifies fractured bone with an accuracy of 92%. Feature extraction is improved with a variety of image processing techniques, such as controllable filters, image projection integration, and image pixel intensity [10]. All of these methods have done a tremendous job for bone diagnosis. However, the performance of these methods is not optimal due to the handcrafted features used for the training of the model. 

Recently, several researchers reported that merging the deep CNN model to form a pool of features improves the overall classification performance. Recently, Lin et al. [11] developed fracture R-CNN (Region-based Convolutional Neural Network) for skull fracture detection. They employed prior clinical knowledge in faster R-CNN to enhance the classification performance. Kitamura et al. [12] developed an ensemble-based deep CNN model for ankle fracture detection. Their method includes Xception, InceptionV3 and ResNet for feature extraction. The ensemble-based model classifies healthy and fractures with an accuracy of 81%. In the transfer learning-based approach, Choi et al. [13] applied YOLOV3 model for skull fracture identification and achieved a sensitivity of 91.7%. In similar research, Kitamura et al. [14] and Kim et al. [15] applied pre-trained DenseNet-121 and InceptionV3 models for bone diagnosis. The method described in [14] achieved 95% accuracy, whereas the method described in [15] achieved an accuracy of 95.4%. 

Some research reported that they first trained their model on a bone image dataset before classification is performed. In this regard, Yang et al. [16] developed two deep CNN models for intertrochanteric fracture segmentation and identification. They split the dataset into two parts, i.e., training and testing, in which 32,045 and 11,465 images are used, respectively. First, the region of interest (ROI) is extracted using cascade architecture-based CNN. After that, another CNN is used for segmentation and identification. In other research, Haitaamar et al. [17] performed preprocessing on a rib fracture image to resize the image into 128 × 128 × 333 pixels. After that, a semantic segmentation technique was applied to locate the fractured region of the ribs. Finally, the UNet model was used to classify CT scan images and achieved an accuracy of 88.54%. Nguyen et al. [18] applied the Yolo 4 model for the localization of fractured bone. The data augmentation technique was also applied and their model performance was compared with original and augmented datasets. On an original dataset, this method can differentiate fracture and healthy regions with an accuracy of 81.94%. A pyramid network was designed by Wang et al. [19] for bone diagnosis. They trained the pyramid network on an X-ray image. Their method achieved an accuracy of 88.7%. Ma et al. [20] identified bone fracture in two steps; in the first step faster R-CNN was utilized to identify 20 fracture regions and in the second step, a novel CrackNet for bone fractured classification. Their method classifies healthy and fractured bone with a classification accuracy of 90.14%. In similar research, a new Parallel Net approach for classifying fractured bone has been proposed using the two-scale method proposed by Wang et al. [21].

Yahalomi et al. [22] and Abbas et al. [23] applied pre-trained faster R-CNN on a small dataset and achieved an accuracy of 96% and 97%, respectively. Luo et al. [24] applied their expert knowledge and designed a decision tree for fractured bone diagnosis. Their method achieved an accuracy of 86.57%. Beyaz et al. [25] and Jones et al. [26] applied deep CNN for the feature extraction from bone images. Their method validation accuracy is 83% and 97.4%, respectively. Dupuis et al. [27] designed the Rayvolve^®^ model for bone fracture detection in children. The external validation method classifies healthy and fractured bone with an accuracy of 95%. Hardalaç et al. [28] applied an ensemble-based deep CNN model for wrist fracture identification. Their method segregates fracture and healthy wrist bone with an accuracy of 86.39%.

Pranata et al. [29] classified calcaneus fractures in CT images using a fusion of the deep features and SURF features. They compared the performance of VGG16 and ResNet, which are two pre-trained deep CNN models. Out of these two models, ResNet achieved the highest classification accuracy of 98%. Mutasa et al. [30] applied the generative adversarial network (GAN) and digitally reconstructed radiographs (DRRs) techniques. The image dataset is created using GAN and classification is performed using a deep CNN model. Weikert et al. [31] applied a deep learning-based CNN model and achieved a classification accuracy of 90.2% for rib fracture identification. Tanzi et al. [32] applied the InceptionV3 model for the classification of proximal femur X-ray images. Their method classifies healthy and fractured femur images with an accuracy of 86%. In similar research, Lotfy et al. [33] applied DenseNet for femur fracture classification and achieved an accuracy of 89%. In [34], the authors have compiled a review work and defined the most important points, what should be taken into account to achieve that goal, and they have compared various models to humans in the classification of fractures. Table 1 provides a summary of the recent research for bone diagnosis.

## 3. Existing Methods

In the proposed research, we have analyzed the performance of AlexNet, MobileNetV2, ResNeXt and VGG16. 

### 3.1. AlexNet Model

**Figure 1:**
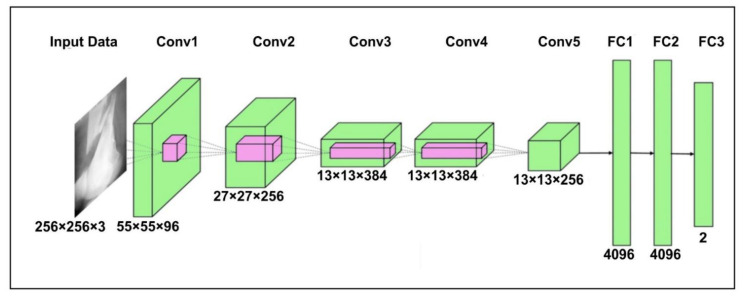
The AlexNet model architecture for bone diagnosis.

The AlexNet [35] model contains three dense layers and five convolutional layers. The first step is to scale the bone image to 256 × 256 pixels, corresponding to width and height, and a three-color channel that represents the depth of the image. The output of neurons is calculated using their respective weights as the inner product of a small portion of the image. First convolution layer (Conv1) size is fixed to 96 and a filter of size 11 × 11 with steps of four is added. After that, the output of the first convolutional layer is fed to the second convolution layer (Conv2) of 256 and filter of size 5 × 5. The third (Conv3) and fourth layers (Conv4) have 384 kernels with filters of 3 × 3. Finally, the last convolutional layers have a kernel of 256 and a filter of size 3 × 3. A m-pooling of 2 × 2 is applied after each convolution layer. The FC1 and FC2 are the fully connected layer search with 4096 neurons. The FC3 uses the Softmax function to classify bone into healthy and fracture. The detailed architecture of the AlexNet for bone diagnosis is shown in Figure 1.

### 3.2. MobileNetV2 Model

**Figure 2:**
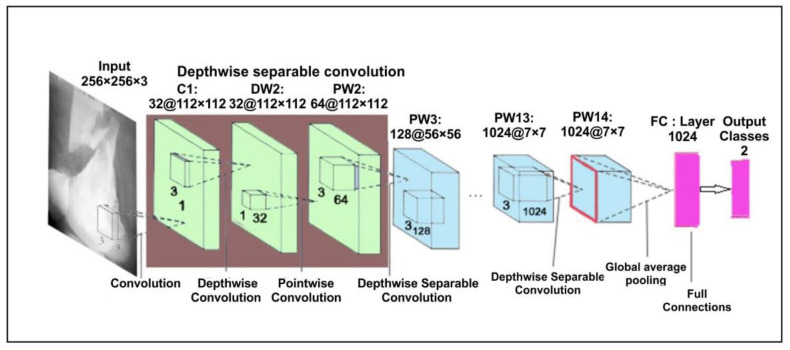
MobileNetV2 architecture for bone diagnosis.

In the proposed approach, we have used pre-trained MobileNetV2 [36] architecture trained on ImageNet. This model is a lightweight network and the significant aim is to minimize the parameters so that the computation is done quickly. To minimize the parameters, it uses a separate layer of convolution. In addition, MobileNetV2 uses depth-wise separable convolutions that are supportable in a very effective manner for a smartphone [37]. A depth-separable convolution layer is the combination of a point convolution layer and a deep convolution layer. At each input path, the deep convolution layer performs a single convolution. After that, the point convolution layer compares the output of the deep convolution layer. It works on a new communication mode that would link each of the current network layers to the previous network layers; with fewer convolution kernels, it can use the previous features repeatedly to create more feature maps. The basic structure has a growth rate of four with four tightly connected layers. Each layer considers input from its previous layer’s output in the form of a feature map in this system. The architecture of the MobileNetV2 is shown in Figure 2.

### 3.3. ResNeXt Model

**Figure 3:**
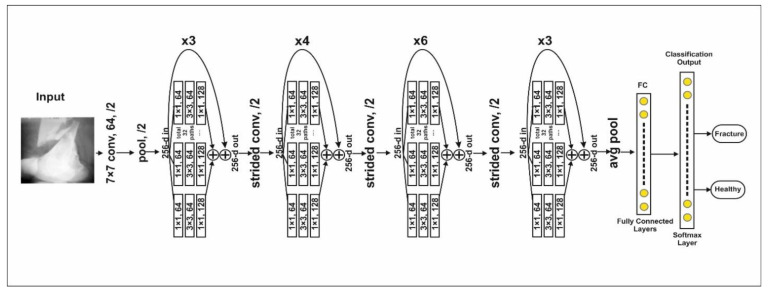
The architecture of the ResNeXt model.

The ResNeXt is a residual class deep CNN model. This model is 50 layers deep and was first runner up in ILSVR 2016 image classification [38]. ResNeXt’s major advantage is a reduction in the number of hyperparameters compared to ResNet-50 using cardinality by adding additional depth and width. The complete architecture is shown in Figure 3. Residual blocks in the model are defined as

(1) $$\alpha =\beta +\sum_{k=1}^{C}F_{k}(\beta )$$

where, C= 32 (cardinality hyper-parameter), β= input parameter vector, α= Output of the model.

### 3.4. The VGG16 Model

**Figure 4:**
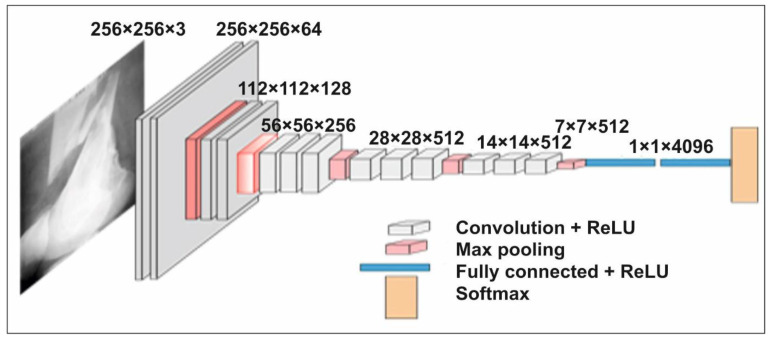
The VGG16 model architecture for bone diagnosis.

The network VGG16 [39] is considered one of the superior architectures of the vision model to date. The strangest thing about VGG16 is that it replaces the huge number of hyper parameters. The VGG16 network is 16 layers deep and has a kernel of size 3 × 3. In addition to this, five layers of this model have max-pooling layers of 2 × 2. After the last max-pooling layer, it has three dense layers, one fully connected layer and Softmax as a classifier layer. The architecture of the VGG16 for the proposed bone diagnosis is shown in Figure 4.

The proposed model can be used for real-time human bone diagnosis by creating web and mobile applications to support a doctor. This model has a smaller number of neurons and computation time per epoch is less compared to the other models discussed in the literature, except MobileNetV2. In addition, classification accuracy is much better than the VGG16, AlexNet, ResNeXt and MobileNetV2. The comparison of each model is shown in Table 2.

## 4. Proposed Hybrid SFNet

In Table 2, we summarized the VGG16, AlexNet, ResNeXt, and MobileNetV2. The VGG16 is a sequential model having 33 × 10^6^ parameters, due to this computation, time is high and overfitting problems may arise. AlexNet is also an eight-layer sequential model with 24 × 10^6^ parameters; this model is less deep due to which it is not able to scan all features. On the other hand, ResNeXt is 50 layers deep with 23 × 10^6^ parameters. Due to this, it has a high training time and a large amount of data are required. MobileNetV2 is 53 layers deep with 6.9 × 10^6^ parameters, this model is lightweight due to its depth-wise convolution architecture, but efficiency is less compared to other state-of-the-art deep CNN models.

**Figure 5:**
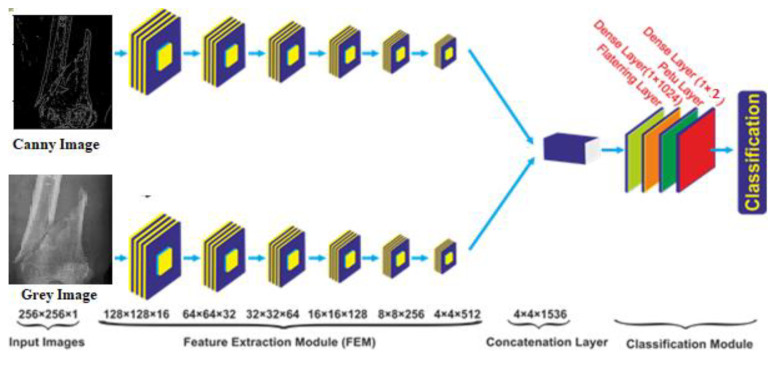
Proposed multi-scale feature fusion CNN model.

In the proposed study, a novel multi-scale hybrid feature fusion model has been developed, which has 5 × 10^6^ parameters with less computation time per epoch compared to other state-of-the-art models discussed above. The complete architecture of the hybrid SFNet is shown in Figure 5. This model contains a two-scale feature extraction model. In each scale, a six-layer convolution module has been utilized. The size of the first, second, third, fourth, fifth and sixth convolution layers is 16, 32, 64, 128, 256 and 512, respectively. Each of the six layers of the feature extraction module contain a rectified linear unit (ReLU) activation, a batch normalization and a global average-pooling layer with a pool size of 2 × 2. The feature extracted from each scale is fused to form a single feature tensor at the concatenation layer. Furthermore, for the classification of bone image, fused tensor is passed to the flattening layer followed by a dense layer of 1024 neurons, ReLU layer, the dropout and the dense layer of two neurons. The classification of bone into healthy and fracture is performed using the Softmax activation function. The Softmax optimizer converts logits into probability. In the present study, feature set *x* and input vector are passed to the system. The class value is determined by setting the value *k* to 2 and class labeling is performed using variable *j*. After that, in each iteration a bias $W_{0}X_{0}$ is added. The mathematical equation is shown in Equation (2).

(2) $$p(y=j|\phi^{\left(i\right)})=\frac{e^{\phi^{\left(i\right)}}}{\sum_{j=0}^{k}e^{\phi_{k}^{\left(i\right)}}}$$

where,

(3) $$\phi =W_{0}X_{0}+W_{1}X_{1}+\ldots +W_{k}X_{k}$$

### 4.1. Local Response Normalization

Local Response Normalization (LRN) improves the ability of excited neurons to suppress adjacent neurons. ReLU neurons have unlimited activation and require an LRN that recognizes high frequency function with a large response. Normalizing the local neighborhood of an excited neuron makes it even more sensitive than that neighborhood. The activity of neurons $\alpha_{u,v}^{j}$ in a place (u,v) by applying the kernel j to the generalization of resources. Then, the non-linearity is applied by ReLU. The $a_{u,v}^{j}$ is the normalized response, which is calculated as per the Equation (4).

(4) $$b_{u,v}^{j}=\alpha_{u,v}^{j}/{\left(t+\alpha \sum_{i-\max(0,j,n/2)}^{\min(N,1,j+n/2)}{\left(\alpha_{u,v}^{i}\right)}^{2}\right)}^{\beta }$$

where, N= Total layers, t,α,n,β = different hyper-parameters. 

The deep CNN model performance depends on the architecture of the models. Therefore, while designing a model, hyper parameters play an important role. The hyper-parameters t,α,n,β are used to enhance the feature map from one layer to another by finding the intensity of the maximum pixels during the local response normalization process. The division by zero is avoided by setting t=2. The consecutive pixels of input layers that undergo normalization are defined by the n. During the normalization process α is set to 10^−4^ and the contrasting constant β is set to 0.75.

### 4.2. Deep Feature Fusion

The feature fusion of two-scale SFNet is defined as follows.

(5) $$A=\{a_{1},a_{2}\ldots \ldots a_{n}\}$$

(6) $$V=\{v_{1},v_{2}\ldots \ldots v_{m}\}$$

where, n=512 and m=512, after that concatenation of these features is calculated as

(7) $$F_{con}=A⊕V=\left(a_{1},a_{2},\ldots a_{n},v_{1},v_{2},\ldots v_{m}\right)$$

where, $F_{con}$ represents the fused feature vector.

### 4.3. Improved Canny Edge Detection Algorithm

The morphological characteristics of fractured bone and healthy bone are different at the fracture location. In addition, the pixel intensity of the fracture zone is less compared to the healthy zone. Therefore, the localization of these pixels needs a method which can detect edges with fewer pixels value suppression. The traditional canny edge detection is widely used for edge extraction in a digital image [40]. However, the traditional method produces isolated edge points due to Gaussian filtering. In addition, it cannot satisfy algorithm adaptability as threshold values are fixed. Furthermore, it also increases the pseudo edge points. It is necessary to address this problem for fracture bone diagnosis, since the pixel intensities at the fracture points are less. Therefore, some changes are made to the traditional canny edge detection algorithm. First, the Gaussian filter is replaced with a median filter. For a particular location of bone, an image pixel value is replaced with a median value. The median filter for a grey image of f(x, y) with filtering window W = M_mxn_ is defined as

(8) $$C(x,y)=Med\{f(x+m,y+n)|M_{mxn}=1,(x,y)\in N\}$$

The adaptive threshold is calculated using the difference of adjacent gradient magnitude, as shown below.

(9) $$G_{1}(i+1)-G_{1}(i)$$

Consider an image with pixel intensity I(x, y)

(10) $$I(x,y)=\left[\begin{array}{l} a_{0}a_{3}a_{6}\\ a_{1}a_{4}a_{7}\\ a_{2}a_{5}a_{8} \end{array}\right]$$

The gradient in x and y direction is calculated as:

(11) $$G_{x}=(a_{6}+2a_{7}+a_{8})-(a_{0}+2a_{3}+a_{6})$$

(12) $$G_{y}=(a_{6}+2a_{7}+a_{8})-(a_{0}+a_{1}+a_{2})$$

The magnitude and direction of the central pixel is calculated as:

(13) $$G_{1}(i,j)=\sqrt{G_{x}^{2}+G_{y}^{2}}$$

(14) θ(i,j)=arctanGxGy

**Figure 6:**
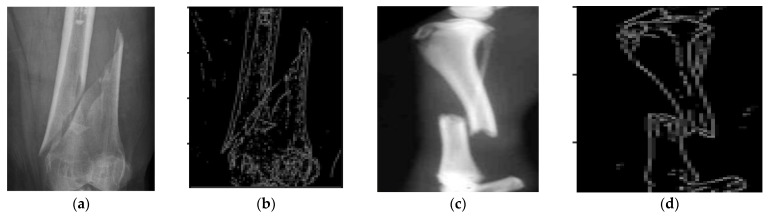
Samples of bone fracture (**a**,**c**) and their corresponding canny (**b**,**d**), respectively.

Samples of bone fracture image is shown in Figure 6a,c and their corresponding canny image in Figure 6b,d, respectively, as shown in Figure 6.

**Figure 7:**
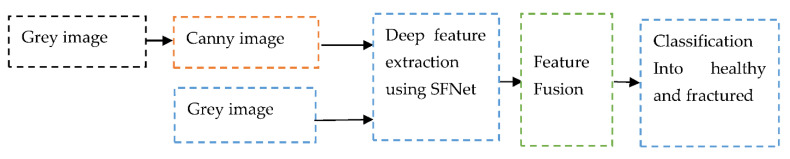
The data flow diagram of the proposed method using hybrid SFNet.

The modified canny edge detection algorithm is able to extract the fractured region. The canny image along with the grey image used for training of the model has produced significant improvement in the classification accuracy. The noise can affect the performance of the deep CNN model. Hence, to obtain a highly robust model, noise effect should be minimized. The improved canny edge algorithm can suppress the noise and produce fine edges of the bone image. That helps the proposed model to learn more accurate features from bone images. Proposed method is depicted in Figure 7.

### 4.4. Loss Function

Loss measures the performance of the model. In order to develop a highly sensitive model, loss should be minimum. In the proposed study, the loss for healthy and fractured bone is calculated as follows.

(15) $$Loss=\frac{1}{N}\sum_{j=1}^{N}-((y_{j}\times \log(P_{j})+(1-y_{j})\times \log(1-P_{j}))$$

where, N = 2, P_j_ = jth scalar value in the model output and y_j_ = corresponding target value. 

## 5. Results Analysis and Discussion

The results obtained using AlexNet, VGG16, ResNeXt, MobileNetV2 and the proposed hybrid SFNet are discussed in this section. In addition, performance measures and their evaluation methods are also explored with the confusion matrices obtained by these models.

### 5.1. Dataset

The Bone X-ray image datasets are obtained from a variety of publicly available sources, such as the Cancer Imaging Archive, The Diagnostic Imaging Dataset (DID) [41,42] and Technology, Shibpur (IIEST) [43].

### 5.2. Performance Parameters

The performance measures of the models are calculated using precision, recall, F1-score and accuracy. These parameters are evaluated from the confusion matrix of each model using the mathematical formula shown in Table 3.

The proposed method is implemented using Python 3.6, Tensorflow2, on TITAN X GPU Nvidia GeForce GTX with 128GB RAM, dual graphics card of 8 GB and window 10 operating system. The initial learning rate 3 × 10^−3^ batch size is 32 and 20 epochs is set for the training of the model.

### 5.3. Result Analysis

The data augmentation technique is also performed to improve the size of the dataset [44]. The basic rotation, flip horizontal, flip vertical and scaling are applied. After data augmentation, the dataset contains 34,000 bone images. Finally, the dataset is randomly divided into training and validation using 80% and 20% ratios, respectively. In this way, each class contain 13,600 images for training and 3400 images for testing. We have maintained the same size 256 × 256 × 3 images for each of the models during training and testing. Each model is trained for 20 epochs with a batch of 32 images. In addition, Adam and RMS Prop Optimizer work with a 3 × 10^−3^ learning rate for the training of models.

**Figure 8:**
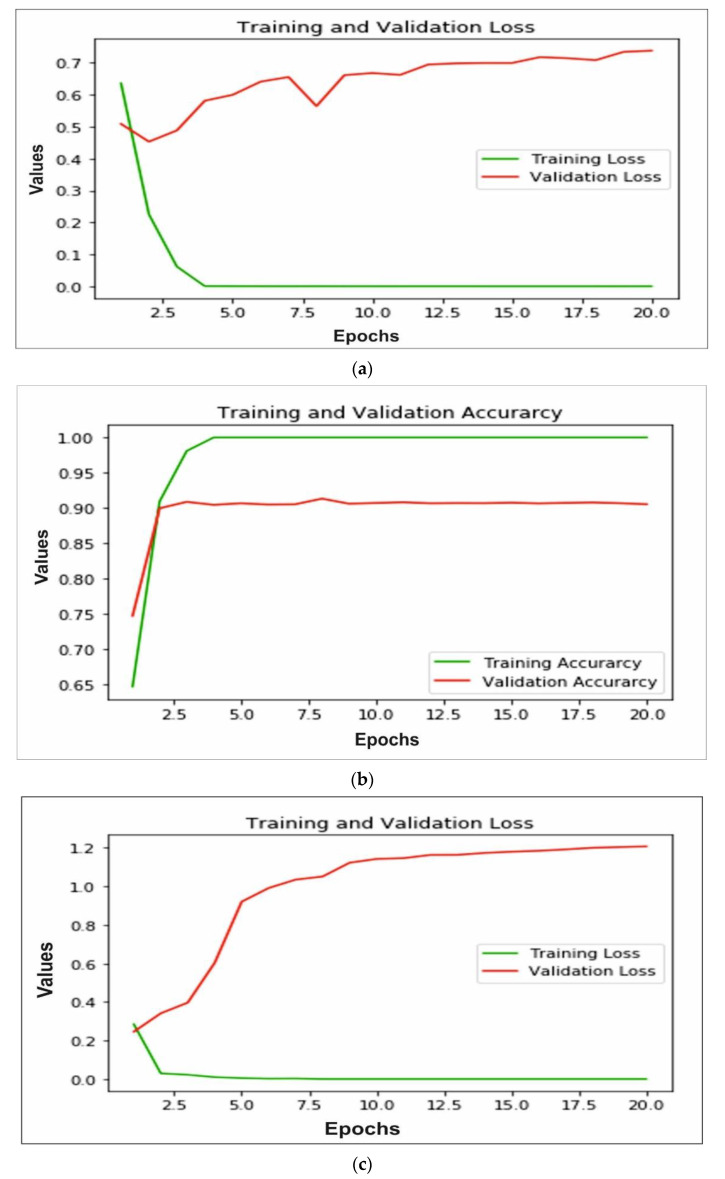

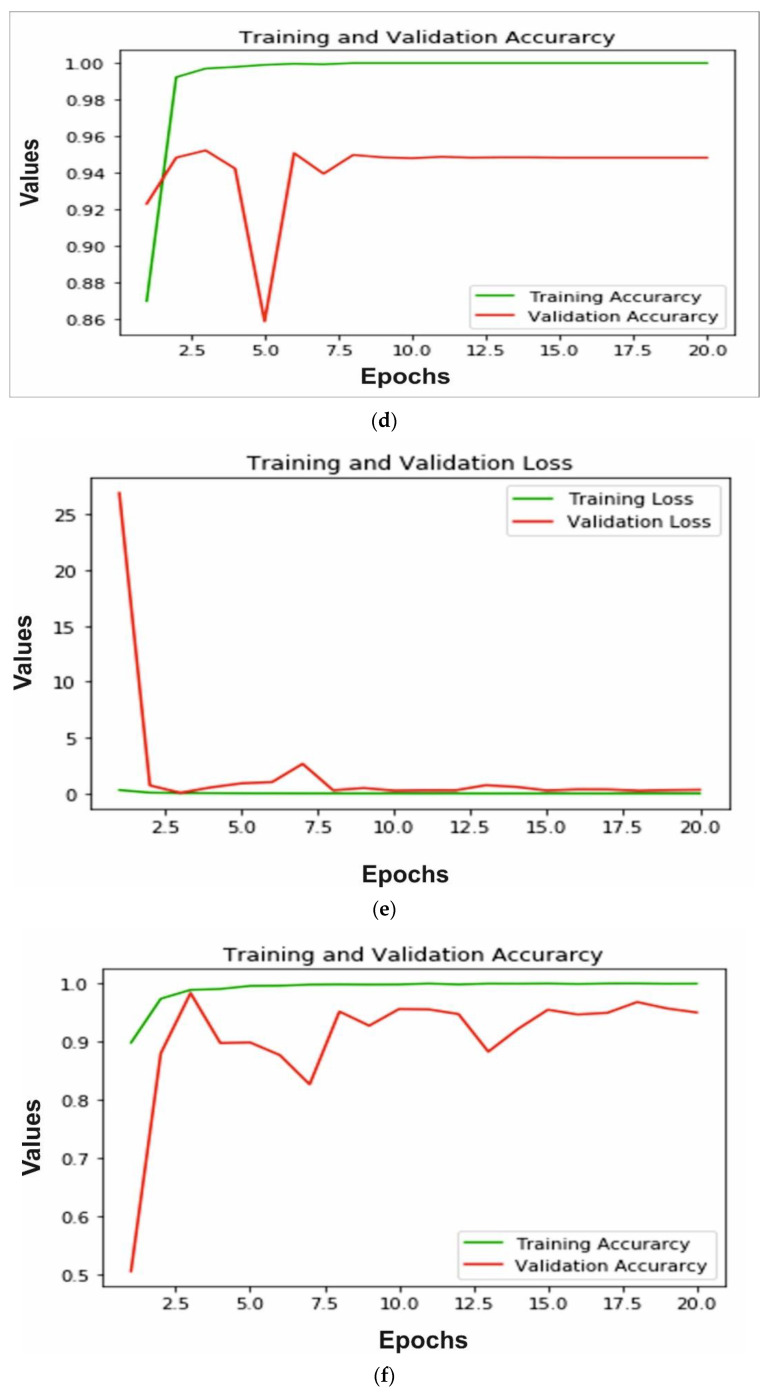

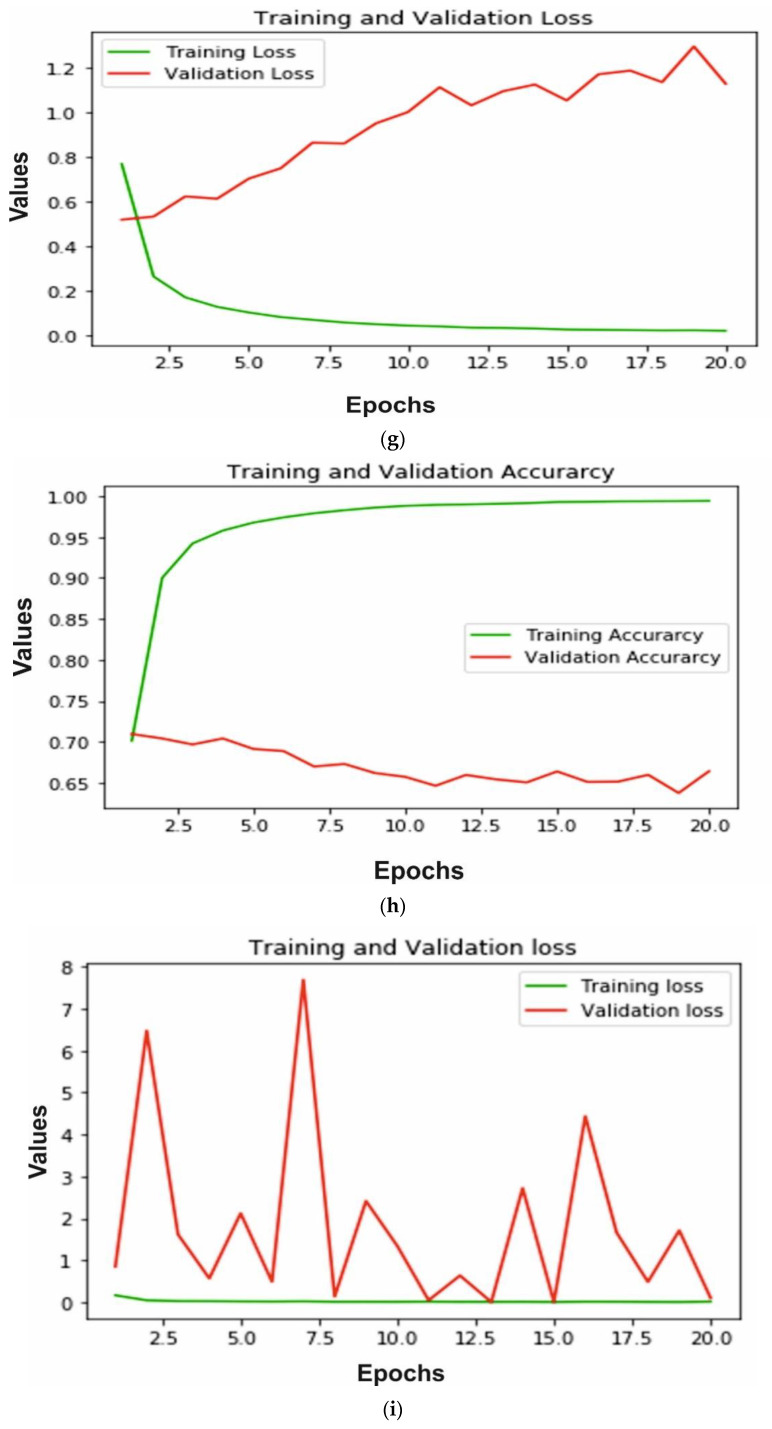

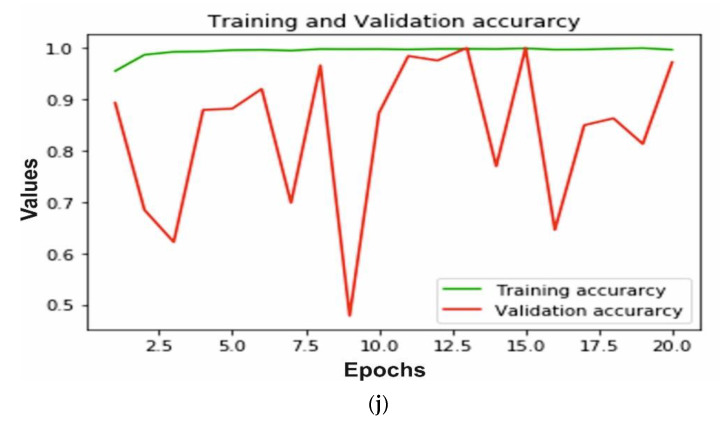
Illustration of training and validation loss of AlexNet, VGG16, ResNeXt, MobileNetV2 and proposed model through (**a**,**c**,**e**,**g**,**i**), respectively, and training and validation loss of AlexNet, VGG16, ResNeXt, MobileNetV2 and proposed model through (**b**,**d**,**f**,**h**,**j**), respectively.

The accuracy and loss of training and validation for each model is calculated to measure the performance. In Figure 8a,b we can observe that for the AlexNet, training loss is very small, whereas the testing loss is increasing; also training accuracy is close to 99% after 10 epochs and testing accuracy decreases after 15 epochs and reaches 90%. A similar pattern can be observed in Figure 8c,d of the VGG16 model. The training loss is very close to 0 and testing loss increases after 10 epochs to reach 1.2. The training accuracy reached 99% and testing accuracy remain close to 94% after 10 epochs. The ResNeXt model loss and accuracy are shown in Figure 8e,f, respectively. The validation and training loss is nearby 0 after 8 epochs and training accuracy reaches 100%, whereas testing accuracy reached 95%. Furthermore, MobileNetV2 trained on ImageNet is implemented for bone diagnosis. In Figure 8g, the training and validation loss of the transfer learning-based approach is depicted. We can observe that the training loss is close to 0, whereas the testing loss increases and reaches 1.2. In addition, the accuracy of training and validation is shown in Figure 8h; the training accuracy reached 99% after 10 epochs and testing accuracy decreases from 70% to 52%. Finally, in Figure 8i,j we can see, using the proposed multi-scale deep learning model, the training and testing loss reached very close to 0, and accuracy reached more than 99%.

**Figure 9:**
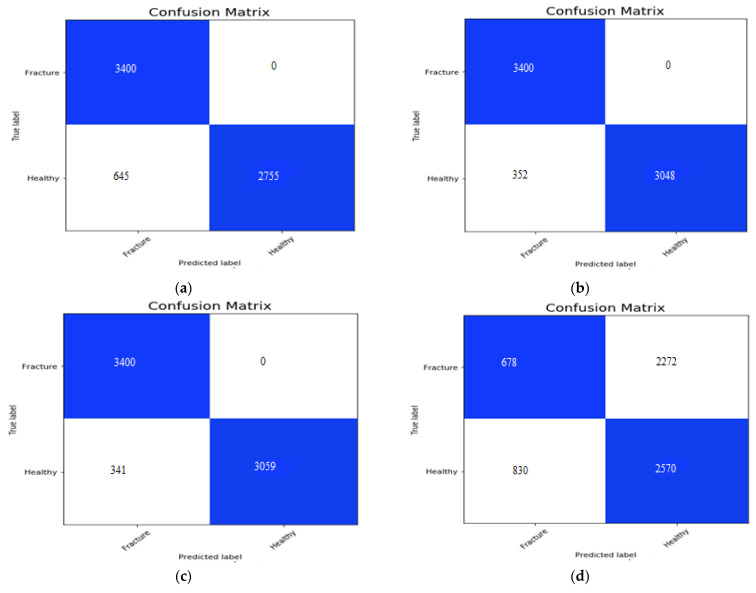

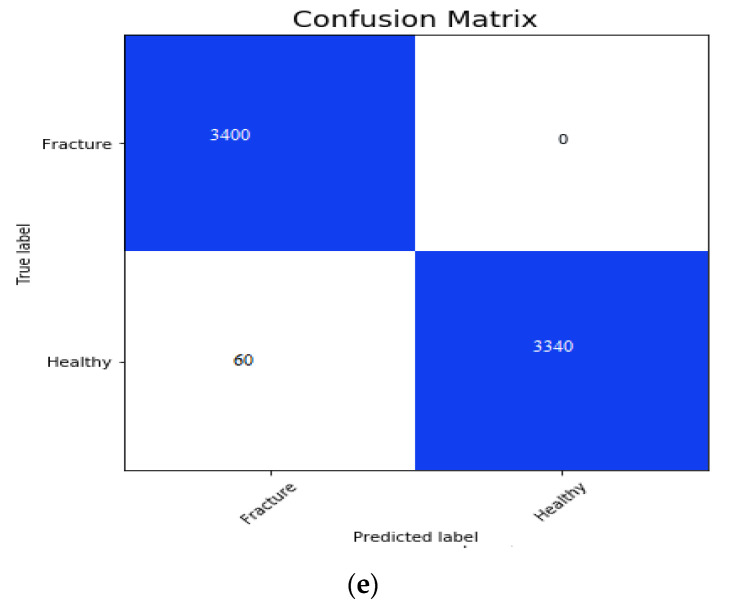
The confusion matrix of the AlexNet, VGG16, ResNeXt, MobileNetV2 and proposed model is shown in the Figures (**a**–**e**), respectively.

The confusion matrix for the AlexNet, VGG16, ResNeXt, MobileNetV2 and the proposed hybrid SFNet is shown in the Figure 9a, Figure 9b, Figure 9c, Figure 9d,e, respectively. The false positive value for the AlexNet, VGG16 and ResNeXt model is 0. However, the false negative value of the AlexNet, VGG16 and ResNeXt is 645, 325 and 341, respectively. The pre-trained MobileNetV2 has the highest false positive and false negative values of 2722 and 830, respectively. In contrast to this, the proposed multimodal has the least number of false negative values of 60. The performance measures of the models are measured using precision, recall, F1-score and accuracy. 

### 5.4. Discussion

The performance measures are shown in Table 4 and confusion matrices obtained in Figure 9. In Figure 9a, the confusion matrix of AlexNet has 3400 TP, 2755 TN, 0 FP and 645 FN values. The confusion matrix of VGG16 is shown in Figure 9b, which has 3400 TP, 3048 TN, 0 FP and 352 FN values. In Figure 9c, we can observe 3400 TP, 3059 TN, 0 FP and 341 FN values yielded by ResNeXt. Furthermore, Figure 9d, shows the confusion matrix of pre-trained MobileNetV2, which has 678 TP, 2570 TN, 2272 FP and 830 FN values. Finally, in Figure 9e the confusion matrix obtained by hybrid SFNet is shown, which has 3400 TP, 3340 TN, 0 FP and 60 FN values. 

In the Table 4, we can see that the precision of the AlexNet, VGG16, ResNeXt and the proposed hybrid SFNet is 100% for the healthy bone. The least value of precision, 49% for the healthy bone, can be seen in the MobileNetV2. Similarly, for fractured bone, the least precision value of 45% was achieved using MobileNetV2 and the highest value of 100% was observed using the proposed hybrid SFNet. The recall value of 100% was observed in fractured bone using AlexNet, VGG16 and ResNeXt, whereas, the proposed model achieved 98% and the least value of 20% was shown by MobileNetV2. In addition, an F1-score of 99% was achieved by the hybrid SFNet, whereas other models reached close to 95%. These results conclude that the performance of the pre-trained MobileNetV2 is not optimal; however, AlexNet, VGG16 and ResNeXt trained on bone image performance is outstanding, but require high computation time and further improvement for fractured bone diagnosis. The proposed hybrid SFNet has shown remarkable performance with the least computation time. 

In addition to this, the recall value of the AlexNet, VGG16, ResNeXt and the proposed model is 100%. However, the highest precision value of 100% is achieved for the fractured bone by the proposed model. Furthermore, the proposed hybrid SFNet model has a classification accuracy of 99% and the other models, such as AlexNet, VGG16, ResNeXt and MobileNetV2, have 91%, 95%, 95% and 48%, respectively.

## 6. Comparative Analysis

A comparative study of the deep learning-based convolution neural network (DCNN) model has been demonstrated. In the past research, some methods are available for the long bone fracture diagnosis. These methods are supportive to a doctor in their diagnosis process, but they are less efficient. Hence, an efficient and robust model for bone diagnosis can be developed, Haitaamar et al. [17] applied U-Net for the segmentation of the bone image. After segmentation, classification is performed with an accuracy of 95%. Nguyen et al. [18] applied YoloV4 for the real-time bone fracture diagnosis and achieved an accuracy of 81.19%. Similarly, Wang et al. [19] developed DCNN for the diagnosis of bone images. Their method confirmed 88.7% classification accuracy.

Wang et al. [20] developed a two-stage deep CNN based method for bone fracture diagnosis and achieved an accuracy of 87.8%. Yahalomi et al. [21] and Abbas et al. [22] applied faster-RCNN for bone diagnosis and achieved an accuracy of 96% and 97%, respectively. Sasidhar et al. [45] evaluated the performance of VGG19, DenseNet121, and DenseNet169. The highest classification accuracy of 92% using DenseNet169 is achieved. All of the methods discussed above are capable of bone diagnosis. However, these methods’ performance is not optimal and real-time application is not available. In the proposed study, we have compared the state-of-the-art deep CNN models AlexNet, VGG16, ResNeXt and MobileNetV2. The performance of the model is evaluated on a publicly available dataset. 

The training dataset size plays an important role to avoid model overfitting. To avoid the overfitting problems, we need to trained the deep CNN model on a large volume dataset. One way to improve the size of the dataset is data augmentation. Therefore, in this research, a data augmentation technique has been applied. 

After data augmentation, the size of the augmented dataset is 34,000. The healthy and fractured bone image contains an equal number of 17,000 images and the dataset is divided randomly into 80% for the training and 20% for the validation. None of the testing images have been included in the training of the model. All simulations are done using Keras library with back-end TensorFlow on window 10 operating system with 128 GB RAM with dual 8 GB graphics. We have taken care that the size of the image will be 256 × 256 for each model used in this study. In addition, each model is trained for 20 epochs with a batch of 32 images. The Adam optimizer has been used for AlexNet, ResNeXt, MobileNetV2, and the proposed multimodal deep learning model with an initial learning rate of 3 × 10^−3^. In the experiment, we found that the Adam optimizer does not produce an optimal performance with the VGG16; flipping to the RMSprop with initial learning of 0.00001 produces a satisfactory performance for the bone diagnosis. 

In addition, Table 5 summarizes the different state-of-the-art methods for bone fraction detection. We can see in Table 5 that hybrid SFNet with only grey image and faster R-CNN have the same classification accuracy of 97%. However, SFNet with grey and canny image performance is highest compared to the state-of-the-art model. This high performance is achieved by the hybrid SFNet model using canny image, due to the localization of fracture region as described in Algorithm 1 and Section 4.3. The features learned by the model from the localized region improves the training and classification accuracy.
**Algorithm 1:** Bone diagnosis technique using hybrid SFNet1: Create a fractured and healthy image dataset. 2: Apply augmentation technique rotation, flip horizontal, flip vertical and scaling to increase the size of the dataset. 3: Find the edge in an image using the improved canny edge algorithm discussed in Section 4.3. 4:  For I = 1 to 20 train the model      (a): Input grey and canny images to hybrid SFNet      (b): Apply Equations (9) and (10) to convert logits into probability values      (c): Calculate training and validation loss for each epoch using equation 155: Find overall training accuracy using the equation discussed in Table 3. 6: Find overall validation accuracy using the equation discussed in Table 3. 7: Find the loss of the hybrid SFNet. 8: Plot a training and validation loss graph for 20 epochs.

**Figure 10:**
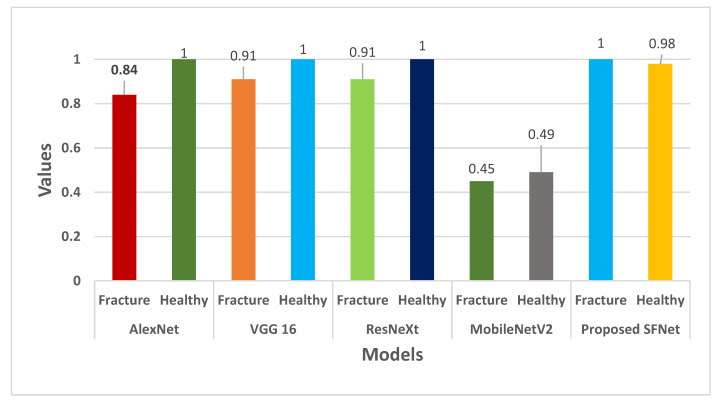
Precision comparison of the state-of-the-art method with hybrid SFNet.

The performance measures precision for each model is shown in Figure 10. In Figure 10, it can be noticed that the highest precision value of 100% for fractured bone is obtained by the hybrid SFNet. The VGG16 and ResNeXt have a similar value of 91%. Whereas, AlexNet showed 84% and the least value of 45% is yielded by the pre-trained MobileNetV2.

**Figure 11:**
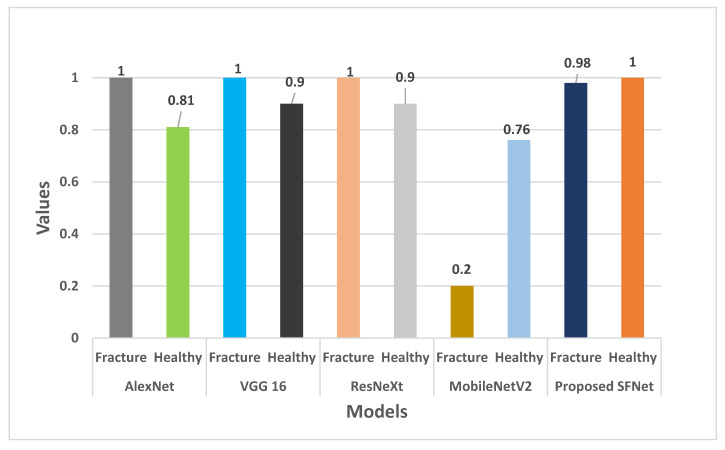
Recall comparison of the state-of-the-art method with hybrid SFNet.

In Figure 11, the recall value of each model is plotted. The recall value of AlexNet, VGG16 and ResNeXt for fractured bone is 100%. Whereas, Hybrid SFNet achieved 98% and MobileNetV2 obtained 20% recall values. For healthy bone, hybrid SFNet has 100% recall value and other models reached 90%, except MobileNetV2.

**Figure 12:**
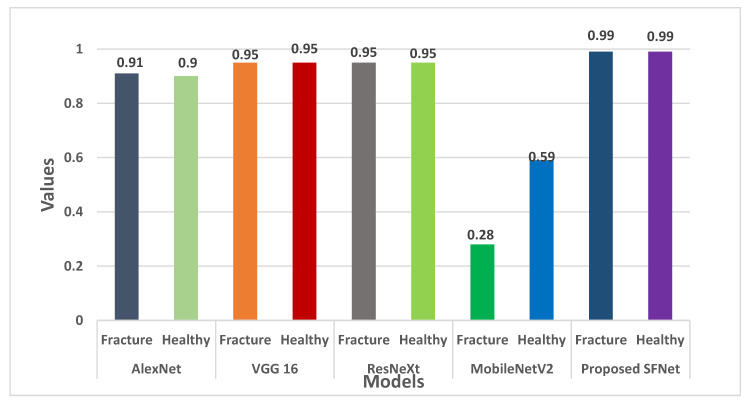
F1-score comparison of the state-of-the-art method with hybrid SFNet.

Furthermore, the F-score for each model is shown in Figure 12. For both fractured and healthy bone, the proposed SFNet achieved a 99% F1-score. Whereas, VGG16 and ResNeXt obtained 95% and AlexNet a 91% F1-score. The least F1-score of 59% for healthy bone was observed in MobileNetV2.

**Figure 13:**
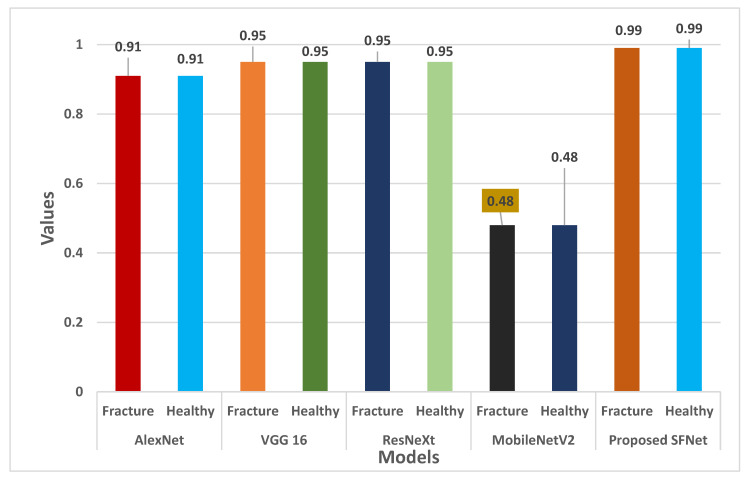
Accuracy comparison of the state-of-the-art method with hybrid SFNet.

Finally, classification accuracy of each model is shown in Figure 13. In Figure 13, we can notice that the highest classification of more than 99% is obtained by hybrid SFNet. The VGG16 and ResNeXt achieved remarkable accuracy of 95%. The AlexNet and MobileNetV2 performance is not optimal. 

We can conclude from Figure 10, Figure 11, Figure 12 and Figure 13 that the performance measures of the proposed SFNet is highest as compared to the state-of-the-art deep CNN model. These performance measures indicate that the proposed hybrid SFNet deep learning model are supportive to a doctor to take a second opinion on the fracture and healthy bone diagnosis. 

## 7. Conclusions and Future Work

In the present work, we have proposed a novel multimodal feature fusion hybrid SFNet. The SFNet has less parameters compared to AlexNet, VGG16 and ResNeXt. In addition, it takes less computation time per epochs, as discussed in Table 2. The grey image and their corresponding canny image are fed to the hybrid SFNet for deep feature extraction. After feature extraction, a pool of features is passed to the classification module. The classification of healthy and fractured images is performed using the Softmax layer. Furthermore, the performance measures by F1-score, precision and recall for the fractured bone as well as healthy bone are calculated. The achieved accuracy of 99.12% and precision of 99% by the hybrid SFNet indicates that canny image improves the performance. Furthermore, the training and validation losses are close to 0. This confirms the model is highly sensitive.

In future studies, we will locate the size of the fracture region, which will help experts to take more action related to bone fracture. In addition, algorithm computation cost will be reduced using optimization techniques. Cloud computing and machine learning play a crucial role in illness detection, but mostly among those who live in distant places with few medical services. While cloud-based systems can enable telehealth services and remote diagnostics, diagnosis tools based on machine learning operate as secondary readers and help radiologists in the accurate diagnosis of illnesses [46]. Due to its capacity to deal with uncertainty and imprecision, neutrosophic theory is frequently employed to solve challenges in medical image processing. Due to the presence of fuzziness and the constrained subjectivity of the specialists utilizing medical pictures, diagnosis is one of the challenging jobs [47]. Image segmentation is one of the most difficult procedures due to specialists’ limited observation and uncertainty in medical understanding. Because imprecise information is commonly employed in decision making, crisp values are insufficient to simulate real-world situations. The intuitive set is a helpful tool for describing images with unclear information [48,49].

## Figures and Tables

**Table 1:** Summary of recent research for bone diagnosis.

Study	Type of Image	Feature Extraction Model	Dataset	Accuracy
Kitamura et al. [14]	X-ray	DenseNet-121	14,374 images	95%
Kim et al. [15]	X-ray	Pretrained InceptionV3	11,112 images	95.4%
Yang et al. [16]	CT Scan	CNN	43,510 images	89.4%
Haitaamar et al. [17]	CT Scan	UNet	150 images	88.54%
Nguyen et al. [18]	X-ray	YoLo4	4405 images	81.91%
Wang et al. [19]	X-ray	Pyramid Network	3842 images	88.7%
Ma et al. [20]	X-ray	CrackNet	1052 images	90.14%
Wang et al. [21]	X-ray	ParallelNet	3842 images	87.8%
Yahalomi et al. [22]	X-ray	Faster R-CNN	38 images	96%
Abbas et al. [23]	X-ray	Faster R-CNN	50 images	97%
Luo et al. [24]	X-ray	Medical decision trees	1000 images	86.57%
Beyaz et al. [25]	X-ray	Deep CNN	2106 images	83%
Jones et al. [26]	X-ray	Deep CNN	715,343 images	97.4%
Dupuis et al. [27]	X-ray	Rayvolve^®^	5865 images	95%
Hardalaç et al. [28]	X-ray	Ensembles deep CNN	569 images	86.39%
Pranata et al. [29]	CT Scan	ResNet + VGG16 + SURF	1931 images	98%
Mutasa et al. [30]	X-ray	GAN + DRS	9063 images	96%
Weikert et al. [31]	CT Scan	Deep CNN	511 images	90.2%
Tanzi et al. [32]	X-ray	Inception V3	2453 images	86%
Lotfy et al. [33]	X-ray	DenseNet	1347 images	89%

**Table 2:** Comparison of different deep CNN model.

Model	Parameters	Time per Epoch	Limitations
VGG16	33 × 10^6^	168 s	This model has a high number of parameters due to long training time
AlexNet	24 × 10^6^	115 s	The performance of the model is not optimal since it is not very deep and it struggles to scan all features.
ResNeXt	23 × 10^6^	140 s	This model is 50 layers deep and requires more training time. Hence, difficult to implement for real-time applications.
MobileNetV2	6.9 × 10^6^	112 s	MobileNet is small in size, small in parameters, and fast in performance. It is less accurate than other state-of-the-art networks.

**Table 3:** The mathematical formula to calculate performance measures.

Measures	Formula	Definition
Accuracy	$\mathrm{Accuracy}=\left(\frac{TP+TN}{TP+TN+FP+FN}\right)$	It is calculated by the ratio of the total number of correctly predicted to the total number of test images.
Precision	$\mathrm{Precision}=\left(\frac{TP}{TP+FP}\right)$	The precision is calculated using actual results divided by the total number of true positive samples.
Recall	$\mathrm{Recall}=\left(\frac{TP}{TP+FN}\right)$	The recall is calculated using the total number of positive samples relative to the total number of predictions.
F1-score	$F1-\mathrm{Score}=2\times \frac{\mathrm{Precision}\times \mathrm{Recall}}{\mathrm{Precision}+\mathrm{Recall}}$	The F1-score measures the harmonic mean of the model performance.

**Table 4:** The comparison of performance measures.

Model	Types of Bone	Precision	Recall	F1-Score	Accuracy
AlexNet	Fracture	0.84	1	0.91	0.91
	Healthy	1	0.81	0.90	
VGG 16	Fracture	0.91	1	0.95	0.95
	Healthy	1	0.90	0.95	
ResNeXt	Fracture	0.91	1	0.95	0.95
	Healthy	1	0.90	0.95	
MobileNetV2	Fracture	0.45	0.20	0.28	0.48
	Healthy	0.49	0.76	0.59	
Proposed hybrid SFNet	Fracture	1	0.98	0.99	0.99
	Healthy	0.98	1	0.99	

**Table 5:** Comparison table based on accuracy of the models.

Study	Model	Accuracy
Haitaamar et al. [17]	U-Net	95%
Nguyen et al. [18]	YOLOv4	81.91%
Wang et al. [19]	DCNN	88.7%
Ma et al. [20]	Faster R-CNN	90.11
Wang et al. [21]	Two-stage R-CNN	87.8%
Yahalomi et al. [22]	Faster R-CNN	96%
Abbas et al. [23]	Faster R-CNN	97%
Sasidhar et al. [45]	VGG19,DenseNet121, DenseNet169	92%
Proposed method	Hybrid SFNet + Grey	97%
	Hybrid SFNet + Canny + Grey	99.12%

## Data Availability

There are no available data to be stated.
